# What helps or hinders the communication of poor prognosis between secondary and primary care? A systematic review with narrative synthesis

**DOI:** 10.3399/BJGP.2023.0341

**Published:** 2024-11-12

**Authors:** Lucy Pocock, Tanuka Palit, Adam McDermott, Sam Creavin, Emma Gilbert, Samuel WD Merriel, Steven Moore, Sarah Purdy, Stephen Barclay, Lucy E Selman

**Affiliations:** Bristol Medical School, University of Bristol, Bristol.; Bristol Medical School, University of Bristol, Bristol.; Bristol Medical School, University of Bristol, Bristol.; Bristol Medical School, University of Bristol, Bristol.; Bristol Medical School, University of Bristol, Bristol.; Centre for Primary Care and Health Services Research, University of Manchester, Manchester.; e, North Bristol NHS Trust, Bristol.; Bristol Medical School, University of Bristol, Bristol.; Primary Care Unit, Department of Public Health and Primary Care, University of Cambridge, Cambridge, UK.; Bristol Medical School, University of Bristol, Bristol.

**Keywords:** advance care planning, communication, continuity of patient care, primary health care, prognosis, secondary care

## Abstract

**Background:**

The communication of poor prognosis from secondary to primary care helps to ensure that patients with life-limiting illness receive appropriate coordinated care in line with their preferences. However, little is known about this information-sharing process.

**Aim:**

To determine how poor prognosis is communicated from secondary care to primary care.

**Design and setting:**

This was an international systematic review and narrative synthesis of studies published in English.

**Method:**

Four electronic databases were searched from 1 January 2000 to 17 May 2021, supplemented by hand-searching of key journals. One-quarter of titles and abstracts were independently screened by a second reviewer. Two reviewers undertook data extraction and quality appraisal, independently using the Mixed Methods Appraisal Tool. Data were analysed using narrative synthesis. Reporting follows Preferred Reporting Items for Systematic Reviews and Meta-Analyses guidance.

**Results:**

Searches identified 23 853 unique studies of which 30 met the inclusion criteria. Few studies had a focus on the interprofessional communication of poor prognosis. Information about prognosis was not commonly communicated from secondary to primary care and was more likely to occur if death was imminent. Lack of identification of poor prognosis by secondary care teams was a barrier. Facilitators included shared electronic records and direct clinician–clinician contact. GPs welcomed this information from secondary care and felt it was vital for continuity of care.

**Conclusion:**

Although the communication of poor prognosis from secondary to primary care is highly valued it is rare and associated with cultural and systemic challenges. Further research is necessary to understand the information needs of GPs and to explore the challenges facing secondary care clinicians initiating this communication.

## Introduction

Sharing information between secondary care (hospitals and specialists) and primary care (GPs and community health services) allows for informational continuity.1 This can help identify gaps in care that need addressing and facilitate care coordination between providers, leading to better health outcomes and experiences for patients.

Continuity of care is particularly important for people towards the end of life because it can help ensure that their care needs are met in a comprehensive and coordinated manner.^2^^,^^3^ When continuity of care is in place, patients are more likely to receive consistent and appropriate care in their preferred place. Strategies that improve continuity of care can reduce hospital deaths and emergency admissions in the last weeks of life, potentially improving the quality of end-of-life care while reducing cost.^4^^,^^5^

Poor communication between professionals hinders the delivery of palliative care by primary care teams.^6^ A lack of communication from secondary care in relation to prognosis, patients’ previously expressed wishes, and limitations to their treatment is a potential barrier to the initiation or continuation of advance care planning (ACP) conversations by GPs; addressing these information needs would facilitate this process.^7^^–^9

Information is shared from secondary care to primary care, predominantly in written forms such as clinic letters and discharge summaries. It is unclear how information about prognosis and ACP is communicated between these settings.

The aim of this review was, therefore, to determine how poor prognosis is communicated from secondary care to primary care. The review questions were as follows:

- How is poor prognosis communicated from secondary care to primary care?
- What are the facilitators of, and barriers to, this communication?
- How acceptable and useful is this communication to patients, family/carers, and clinicians?
- What is the impact of this communication on patient care?

**Table:** How this fits in

Continuity of care in the last year of life is a concern for patients, families, and clinicians and poor continuity can result in suboptimal care towards the end of life. Little is known about how information on prognosis is communicated from secondary care to primary care. This review found that this communication does not routinely occur, but that primary care clinicians value this information sharing and use it to improve their patient and family care. The authors of the current study suggest that a review by the palliative care team during an admission should always be communicated in the discharge summary and that clinicians working in secondary care should be encouraged to share information regarding prognosis with their primary care colleagues. Further research is required to understand why this information is not shared more frequently, what the information needs of primary care clinicians are, and to capture patients’ views and experiences of this communication.

## Method

The review protocol was conducted and reported with reference to Preferred Reporting Items for Systematic Reviews and Meta-Analyses Protocols (PRISMA-P),^10^^,^^11^ prospectively registered with PROSPERO (CRD42021236087) and published open access.^12^

### Eligibility criteria

Inclusion and exclusion criteria are summarised in Box 1. Following an exploratory database search, which identified fewer than 30 studies that were diverse in research design and context and that included a mix of qualitative and/or quantitative findings, it was decided to include all study types in the synthesis.

**Box 1.** Inclusion and exclusion criteria

**Item**	**Inclusion criteria**	**Exclusion criteria**
Types of participants	Healthcare professionals working in secondary care (including mental health settings and acute hospitals) settings and/or in primary care (general practice)	Clinicians not working in secondary or primary care
	Patients with incurable, advanced disease who have a poor prognosis (likely to be in the last year of life), regardless of age	Patients, or carers of patients, who are not thought to be in the last year of life, including cancer survivors (people whose cancer is in remission and who are no longer being treated) and people with chronic but not life-limiting conditions, for example, diabetes, arthritis
	Current or bereaved carers of people with incurable, advanced disease	Patients who may have a poor prognosis, but where this has not been identified or communicated
Communication or intervention	Studies reporting any type of communication or intervention that facilitates the sharing of poor prognosis from secondary care to primary care. Examples include discharge summaries, clinic letters and shared electronic health records	Studies reporting general communication from secondary care to primary care, without an emphasis on the sharing of poor prognosis
		Studies reporting communication solely from primary care to secondary care, even if there is an emphasis on the sharing of poor prognosis
Type of study	Any study reporting original, empirical data, regardless of study design	Case reports, protocols, editorials, or commentaries
Language of study report	English	Not reported in English
Timeframe	Studies published on, or after, 1 January 2000	Studies published before 2000

### Search strategy

The Medline search strategy (Supplementary Information S1) was developed in collaboration with a professional librarian and adapted for each database (Embase in Ovid, CINAHL, and the Social Sciences Citation Index).

All databases were initially searched from 1 January 2000 to 17 May 2021 and then re-run on 24 August 2022 to include more recently published studies. In addition, *Palliative Medicine*, *British Medical Journal Supportive and Palliative Care*, *British Journal of General Practice*, *European Journal of Cancer Care*, and *Family Practice* were hand-searched from January 2000 to January 2022. Reference and citation searches of all included studies were undertaken.

**Figure 1.**
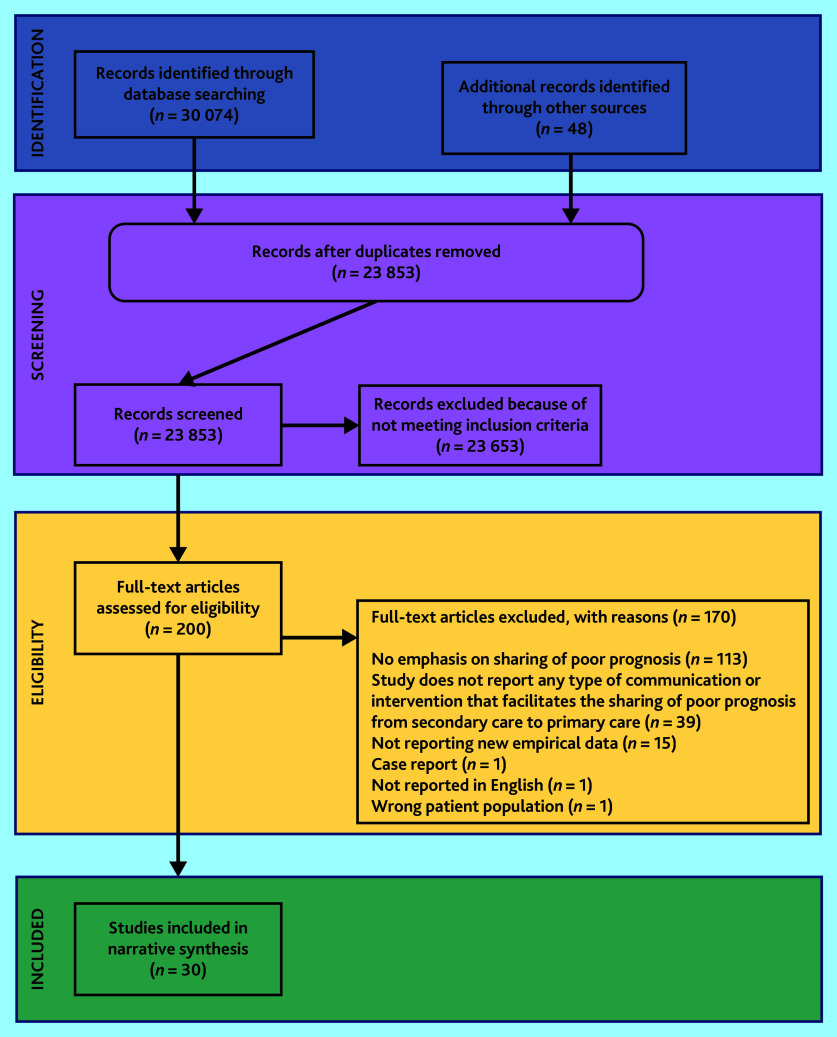
PRISMA flow diagram.

Search results were imported to Covidence^13^ and de-duplicated. The PRISMA flow diagram for study selection is shown in Figure 1.

### Study selection and data extraction

Title and abstract screening were undertaken in Covidence by the first author, with a random 25% independently screened by a second author. There were no conflicts at this stage. Full texts of potentially eligible publications were assessed by the first author.

If study reports were only available as a conference abstract or short report, attempts were made to contact authors to request additional information and further data.

Data were extracted by the first author and independently by one other author, into a review-specific data extraction form within Covidence.

### Quality appraisal

The Mixed Methods Appraisal Tool (MMAT) version 2018 was used to assess the quality and relevance of included publications.^14^ This tool critically appraises quantitative, qualitative, and mixed-methods studies included in mixed-studies reviews. Two screening questions (Are there clear research questions? Do the collected data allow one to address the research questions?) are applied to all studies, following which the type of study design is determined (qualitative, quantitative randomised controlled trials, quantitative descriptive, or mixed methods) and corresponding criteria are used to appraise the quality. The MMAT appraisal was carried out independently by two authors, with disagreements resolved by discussion. Publications assessed as high quality were considered more credible and relevant and were given priority during data synthesis. Given the scarcity and diffuse nature of the evidence identified, the publications assessed as low quality were also included.

### Data synthesis

Data synthesis used a narrative approach, following the framework outlined by Popay *et al.*^15^ Narrative synthesis is ‘an approach to the systematic review and synthesis of findings from multiple studies that relies primarily on the use of words and text to summarise and explain the findings of the synthesis’*.*^15^ This approach was chosen to synthesise the included studies, due to their heterogeneous research design and context, including a mix of qualitative and quantitative findings. Whereas conventional systematic reviews address narrowly focused questions, presenting a summary of the data, narrative syntheses provide interpretation and critique, aiming to deepen understanding.^16^

The narrative synthesis involved the following three iterative stages:

- Developing a preliminary synthesis. The first author created a textual description of each study from the data extraction forms. Study descriptions were grouped together and tabulated based on the research questions the results answered, followed by an inductive thematic analysis to identify the relevant data across the studies in answering each research question.^15^
- Exploring relationships within and between studies. The first author constructed an interpretive synthesis by identifying factors that might explain differences in study findings, seeking to understand how and why interventions have or do not have an effect by interrogating the reported facilitators and barriers to the communication of poor prognosis between secondary care and primary care. The synthesis sought to explore the differences and similarities between the studies, including methodological approaches, context, the characteristics of the populations being studied, and results. The synthesis was further refined through discussion of the review results and their implications with the co-authors, who are from clinical and social science academic backgrounds.
- Assessing the robustness of the synthesis. Methodological quality of the primary studies was assessed by the first author using the MMAT^14^ and integrated into the narrative synthesis, as described above.

### Patient and public involvement

A patient and public involvement advisory panel of seven people with experience of being a current or bereaved carer for someone at the end of life helped to shape the review questions.

## Results

After de-duplication, the electronic searches and hand-searching identified 23 853 individual studies (see Figure 1). A total of 23 653 studies were excluded at title or abstract level. In total, 200 publications were screened at full text, of which 30 met the eligibility criteria and were included in the synthesis.^7^^,^^17^^–^45

### Characteristics of included evidence

The included studies were 24 original articles and six conference abstracts or project reports, using qualitative, quantitative, and mixed methods. Studies were conducted between 2000 and 2022, in: Australia (*n* = 2), Belgium (*n* = 1), Canada (*n* = 3), France (*n* = 1), the Netherlands (*n* = 8), New Zealand (*n* = 1), UK (*n* = 10) and USA (*n* = 4). Supplementary Table S1 summarises the included publications.

### Quality of studies

**Figure 2.**
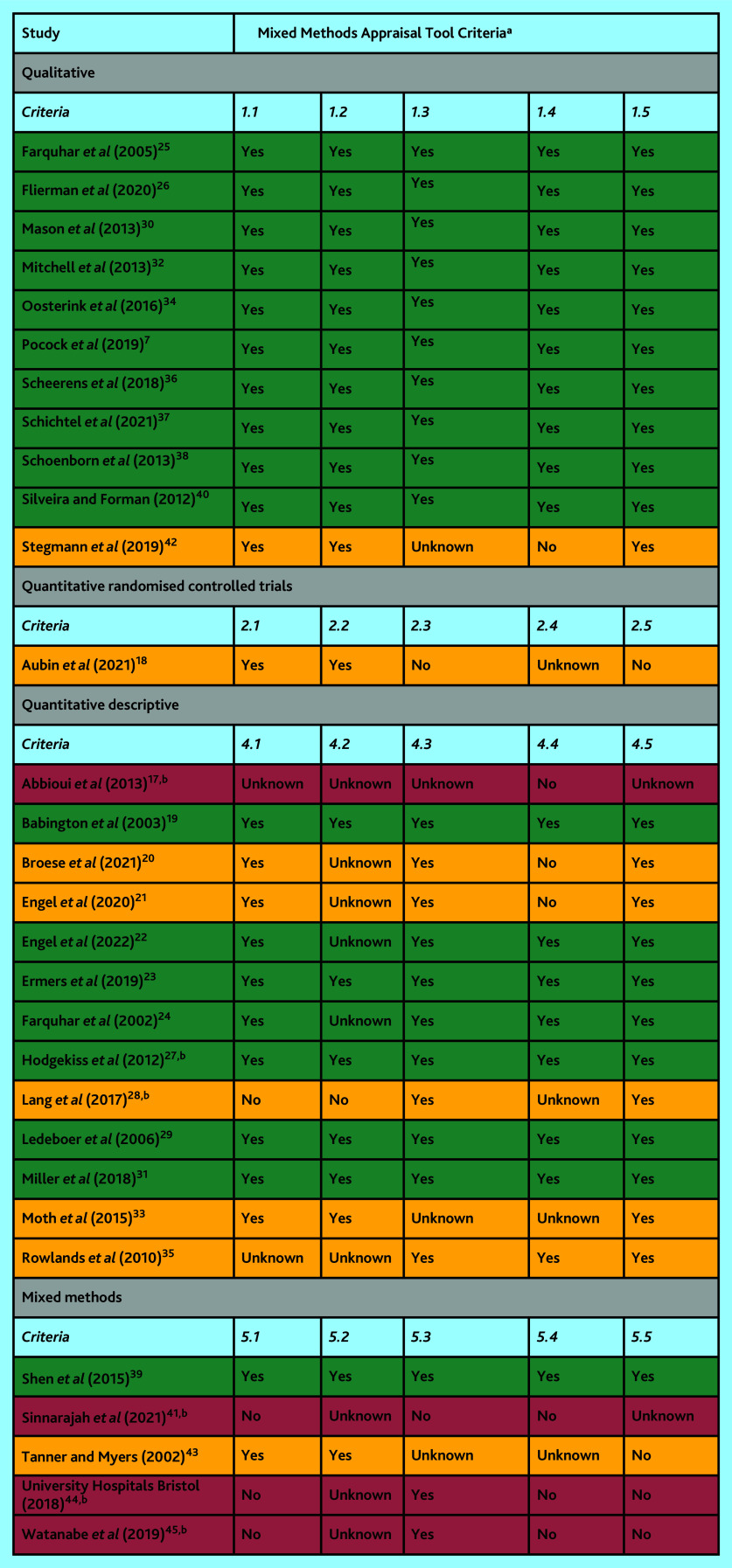
Methodological quality of included studies (high quality in green, medium quality in orange and low quality in red).^a^ Depending on the study type, each criterion asks a question about an aspect of the study, for example, 2.1. Is randomization appropriately performed? Responses can be ‘yes’, ‘no’ or ‘unknown’. The latter response means that the paper does not report appropriate information to answer ‘yes’ or ‘no’. ^b^ Conference abstract or project report.

The methodological quality of the included studies was mixed (Figure 2). Overall, the studies were assessed to have considerable risk of bias, primarily because of insufficient or inadequate reporting. Eighteen of the 30 included studies met all possible appraisal criteria.

### Focus and nature of available evidence

Few studies investigated poor prognosis communication from secondary care to primary care as their primary focus. Most focused on general communication with indirect evidence of poor prognosis communication. Fourteen studies focused on patients with cancer. The number of studies relevant to each review question is presented in Table 1; of the 30 included studies, only five assessed or considered the impact of secondary to primary care communication of poor prognosis on patient care.

**Table 1.** The number of studies addressing each review question

**Review question**	**Studies answering each review question, *n***
**How is poor prognosis communicated from secondary care to primary care?**	25
**What are the facilitators of, and barriers to, this communication?**	14
**How acceptable and useful is this communication to patients, family/carers and clinicians?**	20
**What is the impact of this communication on patient care?**	5

### How is poor prognosis communicated?

The sharing of information about poor prognosis was described in a range of ways including written information following outpatient clinics,^17^^,^^19^^,^^24^^,^^25^^,^^32^^,^^33^^,^^42^ discharge summaries,^22^^,^^24^^–^26^,^^31^ shared electronic records,^23^ telephone^26^^,^^40^^,^^42^ and face-to-face handover.^26^ Two studies evaluated interventions designed for sharing poor prognosis information;^28^^,^^44^ two others evaluated standardised interventions for shared care between oncologists and GPs.^18^^,^^45^

#### Letters

Letters from hospital specialists (usually oncology) to GPs rarely contained information about prognosis or whether treatment intent was curative or palliative. In a high-quality study, Babington *et al*^19^ found that although 72% of patients had a letter sent to their GP after an initial oncology consultation, only 20% of these letters contained any information about prognosis. An Australian high-quality study found that, although the terms ‘metastatic’ or ‘stage IV cancer’ were included for 93% of the 272 included patients with metastatic cancer, the words ‘incurable’ or ‘palliative’ appeared less frequently (34% and 64%, respectively).^33^ A ‘general qualitative remark’ about prognosis was included for 8% patients and a quantitative estimate of prognosis was included for 11% of patients. This was corroborated by Stegmann *et al*,^42^ in a study of medium quality, whose GP participants cited a lack of information about the curative or palliative intent of treatment in letters from hospital specialists.

#### Discharge summaries

The sharing of poor prognosis was not commonplace in hospital discharge communication, even for patients seen by palliative care teams during their admission. A recent, high-quality retrospective review of medical records^22^ found that although information regarding a limited life expectancy was present in the medical record for 70% of all patients, nearly half of discharge letters did not share any information about limited life expectancy. Inclusion of information regarding a poor prognosis was more likely in discharge letters for patients with cancer, but this difference was not statistically significant.

In another high-quality study, 22.8% of discharge summaries for patients who received an inpatient palliative care consultation did not contain any information related to six validated categories, for example palliative care, or plan of care.^31^ This information was more likely to be present the closer the patient was to discharge when the consultation had taken place, and if they were male and older.

#### Telephone

A high-quality American study found that primary care providers would often call a hospital specialist to find out more about the prognosis of their patients.^40^ The GPs and oncology specialists participating in a Dutch study of medium quality mentioned that it was often difficult to contact each other by telephone because of limited availability.^42^

#### Standardised written interventions

Two studies report low-medium-quality evidence on standardised written interventions for facilitating shared care between oncologists and GPs.^18^^,^^45^ Both studies described the sharing of prognosis as one element of the overall intervention, but neither reported on how this particular information had been provided by the oncologist or about how this had an impact on patient care.

#### Poor prognosis letter

The poor prognosis letter (PPL) was a local initiative, developed by the palliative care team in a UK hospital, to be sent by the secondary care team to the GP when discharging patients thought to be in the last year of their life. It tells the GP what conversations have already been had in hospital, so they can support the person going forward, as well as any practical care which is needed. Two low-to-medium quality audits found that, although a minority (38%) of surgical patients with a life expectancy <1 year received PPLs on discharge, 55% of all patients with a poor prognosis in the hospital had a PPL sent.^28^^,^^44^ The median survival following discharge for these patients was 31.5 days.^44^

### What are the facilitators of, and barriers to, this communication?

#### Facilitators

Findings from four high-quality studies suggest that communication of poor prognosis worked well when there was direct clinician-to-clinician contact, either over the phone, or in person.^25^^,^^26^^,^^34^^,^^40^ There is high-quality evidence that sharing of this information was more likely to occur when the patient’s case was clinically complex or they were very sick, if the clinician had a particular concern about them, or if a palliative care review had taken place within the days leading up to discharge.^31^^,^^38^ Clinicians in a high-quality UK study reported improved information sharing when shared registers and electronic records were in use.^30^ A high-quality American study suggested that some providers of primary care proactively called their secondary care colleagues for prognostic information.^40^

#### Barriers

There is medium-high-quality evidence that uncertainty in identifying patients with poor prognosis was a barrier to sharing information, especially for junior doctors.^26^^,^^28^ Nurses in a high-quality Netherlands-based study sometimes had to persuade physicians to provide a statement on life expectancy for insurance coverage, but some felt it should not be included in a written handover.^26^ High-quality evidence suggests that disagreements within clinical teams about prognosis^26^^,^^44^ and a reluctance to encroach on GPs’ professional autonomy were also barriers.^30^^,^^36^ Not receiving any information at all, or receiving it too late, was often cited as a significant impediment to care provision by primary care teams in two high-quality studies.^24^^,^^25^ High-quality evidence also suggests that a lack of standardised communication pathways and an inability to share electronic records across institutions contributed to delayed or no information sharing.^38^^,^^39^

### How acceptable and useful is this communication to patients, family/carers, and clinicians?

High-quality studies report that prognostic information shared by hospital clinicians was useful to GPs in determining the palliative phase for their patients,^20^^,^^32^ initiating ACP conversations,^37^ and knowing how to present treatment options and guide patients though decision making.^40^ In three studies, ranging from low to high quality, GPs reported that prognosis was poorly communicated from secondary care.^7^^,^^17^^,^^42^ Suggested improvements from a high-quality study included less detail about treatment specifics and more about patient coping and management plans.^25^ The views of patients and those close to them were not represented in any of the included studies.

### What is the impact of this communication on patient care?

According to a high-quality study, primary care providers value information about prognosis and a lack of communication can result in discontinuous and lower-quality patient care.^39^ A complex intervention of medium quality, designed to improve interprofessional collaboration, led to better perceptions of collaboration by both patients and family physicians, as well as decreased hospital admissions and emergency department attendances.^18^ Watanabe *et al*^45^ reported in a conference abstract (low-quality evidence) the outcomes of a standardised shared care letter for patients with colorectal cancer, concluding that the letter increased communication and care coordination, although the full study results were not published. Another conference abstract, of medium quality, reported the findings of a local audit of PPLs sent to GPs by surgical teams.^28^ Of 16 PPLs sent, nine patients were added to a GP palliative care register or equivalent and four were directly admitted to a nursing home or hospice. No results were given for the outcomes of patients who did not have a PPL sent.

## Discussion

### Summary

Primary care clinicians highly value receiving prognostic information from their secondary care colleagues and use this to enhance the care they provide for patients. This sharing of information is an example of informational continuity^46^ and allows primary care clinicians to identify patients with palliative care needs. However, the communication of poor prognosis from secondary care to primary care is limited, with a number of barriers including cultural issues around the identification of patients with a limited life expectancy and systemic factors related to information sharing.

### Strengths and limitations

To the authors’ knowledge, this is the first systematic review to explore the sharing of poor prognosis from secondary care to primary care. The search strategies were comprehensive, and the inclusion of both qualitative and quantitative studies provides rich insights into the views on, and experiences of, both primary and secondary care clinicians regarding information-sharing practices and requirements. A transparent and replicable process was achieved through the use of explicitly defined criteria for study selection, systematic methods for data extraction and synthesis, and prospective protocol registration.

Only study reports written in English and published since 2000 were included, so the current findings may not apply in all country settings and are relevant to more recent practice. However, there is evidence that systematic reviews based on a search of English language literature tend to produce similar results to those obtained from reviews without language restrictions.^47^ For pragmatic reasons, full-text screening was only carried out by the first author. This was also justified by the lack of conflict between two independent authors at the title and abstract screening stage. Although the authors acknowledge that conference abstracts have not been subject to the same level of peer-review as published studies, according to Scherer and Saldanha only 37% of studies presented at conferences are published in full and, therefore, ‘restricting a systematic review search to only the published literature would amount to the loss of an immense amount of information’.^48^ This is an area of emerging research, so the inclusion of conference abstracts was also important to capture current and recent work, along with clinical or quality improvement innovations.

The depth of narrative synthesis was limited by the focus of the studies identified and the nature of available evidence, which addressed diverse aspects of information sharing. The views of patients and those close to them were not represented in any of the included studies.

#### Comparison with existing literature

Although the focus of this review is the communication of information from secondary care to primary care, it is clear that improved communication between primary and secondary care, in both directions, is required for well-coordinated care. This is reflected in the national palliative and end-of-life care partnership for England’s six ambitions for palliative and end-of-life care, which highlights the importance of continuity of care, requiring ‘individuals and organisations to think and work in a joined up way so that each is aware and acts in full knowledge of the other’.^49^ The current review echoes the national framework report in highlighting shared records as a potential way to address continuity, allowing bidirectional information sharing between primary and secondary care (along with other care providers). In England, Electronic Palliative Care Coordination Systems (EPaCCS) have been developed to facilitate this cross-organisation information sharing, but these have yet to reach their potential because of difficulties with digital interoperability and poor engagement.^50^

Several of the factors identified to affect the communication of poor prognosis at hospital discharge, such as insufficient or delayed transfer of information, have been previously identified in a systematic review of information transfer at hospital discharge in more general terms, suggesting that these barriers are not limited to the sharing of prognostic information.^51^

Identification of patients who have a limited life expectancy can be challenging. The current review suggests that a lack of appropriate identification and uncertainties about prognosis are barriers to effective communication about patients with a poor prognosis. The gold standards framework proactive identification guidance^52^ has been proposed as an effective screening tool for use in secondary care settings,^53^ although research carried out in England suggests that healthcare professionals struggle to convey the results of this screening to patients and their loved ones.^54^ Etkind and Koffman postulate that prognostic uncertainty can be a source of patient distress, leading to over-investigation and increased healthcare resource use.^55^

### Implications for research and practice

The communication of poor prognosis from secondary to primary care is highly valued, but rarely occurs. One of the main barriers is the identification of patients with a poor prognosis or who have palliative care needs. Hospital teams should be encouraged and supported to identify these patients. A review by the palliative care team during an admission should always be communicated in the discharge summary. Ideally this should be a brief summary of the discussions had with the patient, their family and people close to them, which may include prognosis, the results of ACP conversations, along with advice on symptom control, if appropriate.

Clinicians working in secondary care should be reassured that their primary care colleagues welcome information regarding prognosis and use this information to plan and coordinate care more effectively.

Further research is required to understand the information needs of primary care clinicians in this area and to ensure that the views and experiences of patients and those closest to them are included.

## Data

The data used in this study are previously published papers and reports and are publicly available.
